# Scale-Up of Membrane-Based Zinc Recovery from Spent Pickling Acids of Hot-Dip Galvanizing

**DOI:** 10.3390/membranes10120444

**Published:** 2020-12-21

**Authors:** Andrea Arguillarena, María Margallo, Axel Arruti-Fernández, Javier Pinedo, Pedro Gómez, Ane Urtiaga

**Affiliations:** 1 Chemical and Biomolecular Engineering Department, University of Cantabria, Avda. Los Castros, s.n., 39005 Santander, Spain; andrea.arguillarena@unican.es (A.A.); maria.margallo@unican.es (M.M.); 2 Apria Systems, Parque Empresarial de Morero, Parcela P.2-12, Nave 1-Puerta 5, 39611 Guarnizo, Spain; axel.arruti@apriasystems.es (A.A.-F.); javier.pinedo@apriasystems.es (J.P.); pedro.gomez@apriasystems.es (P.G.)

**Keywords:** hot-dip galvanizing, zinc recovery, spent pickling acid, hollow-fiber membrane contactor, pilot plant, non-dispersive solvent extraction, tributyl phosphate, secondary zinc

## Abstract

Zinc recovery from spent pickling acids (SPAs) can play an important role in achieving a circular economy in the galvanizing industry. This work evaluates the scale-up of membrane-based solvent extraction technology aimed at the selective separation of zinc from industrial SPAs as a purification step prior to zinc electrowinning (EW). The experiments were carried out at a pilot scale treating SPAs batches of 57 to 91 L in a non-dispersive solvent extraction (NDSX) configuration that simultaneously performed the extraction and backextraction steps. The pilot plant was equipped with four hollow fiber contactors and 80 m^2^ of total membrane area, which was approximately 30 times higher than previous bench-scale studies. Tributylphosphate diluted in Shellsol D70 and tap water were used as organic and stripping agents, respectively. Starting with SPAs with high Zn (71.7 ± 4.3 g·L^−1^) and Fe (82.9 ± 5.0 g·L^−1^) content, the NDSX process achieved a stripping phase with 55.7 g Zn·L^−1^ and only 3.2 g Fe·L^−1^. Other minor metals were not transferred, providing the purified zinc stripping with better quality for the next EW step. A series of five consecutive pilot-scale experiments showed the reproducibility of results, which is an indicator of the stability of the organic extractant and its adequate regeneration in the NDSX operation. Zinc mass transfer fluxes were successfully correlated to zinc concentration in the feed SPA phase, together with data extracted from previous laboratory-scale experiments, allowing us to obtain the design parameter that will enable the leap to the industrial scale. Therefore, the results herein presented demonstrate the NDSX technology in an industrially relevant environment equivalent to TRL 6, which is an essential progress to increase zinc metal resources in the galvanizing sector.

## 1. Introduction

The hot-dip galvanizing (HDG) process is one of the most common methods to prevent steel corrosion by providing steel components with a protective zinc coating [1]. Acid pickling is one of the preliminary HDG stages aimed at removing impurities such as oxides from the steel surface [2,3]. It is also used for dezincing of tools and non-conforming galvanized components. At present, HCl is the most commonly used acid for carbon steel pickling, since it provides optimal surface quality and fast pickling [4,5,6].

However, the management of the spent pickling acids (SPAs) constitutes one of the environmental challenges for the galvanizing industry. SPAs after steel pickling in the HDG plants consist of free HCl, iron, zinc, and chloride ions [7]. The present study concerns residual HCl SPAs from the pickling and dezincing of steel items in HDG plants that contain high concentrations of zinc and iron. A freshly prepared pickling bath typically contains 12–16% HCl, although this concentration is progressively reduced along their use [8]. The pickling bath is considered spent when the acid concentration decreases between 75 and 85% of its initial value, and the metals concentration in solution increases to 150–250 g·L^−1^ [6]. It is also worth mentioning that most general galvanizers apply the Kleingarn curve that consists of the periodic replacement of only one part of the pickling bath. This practice allows increasing the pickling rate, reducing the generation of SPAs [4].

Table 1 compiles literature information on the composition of HCl-based SPAs. The iron concentration ranges from 8 to 204 g·L^−1^, the average being 101.6 g·L^−1^. Iron is mostly present as Fe^2+^. Zinc concentration varies in a similar range, the average being 95.7 g·L^−1^. The wide variety of zinc and iron concentration is the result of the diverse practices applied by galvanizers, e.g., the remaining acid can be used for stripping the zinc layer from rejected galvanized steel products [9]. In addition to zinc and iron, SPAs may contain a low concentration of other metals such as manganese, lead, aluminum, chromium, cadmium, nickel, copper, and cobalt [10]. Moreover, SPAs contain surfactants, inhibitors, and stabilizers that may hinder the recovery of acid and/or metals [7].

The conventional SPAs treatment consists of residual acid neutralization with lime or some other cheap alkaline agents [7], which is followed by the disposal of the waste metallic sludge in landfills. Solidification/stabilization can be carried out before the disposal to make the contaminants as immobile as possible. Although neutralization is the most economic method, it presents some disadvantages such as excessive sludge production [26], slow metal precipitation kinetics, inefficient metal removal due to poor settling of metal precipitates, leaching of heavy metals to groundwater, and the problem of landfilling [18]. 

The conventional treatment of SPAs is being substituted by innovative alternatives that can have different objectives: acid recovery, metals recovery, and the conversion of the waste into other products [7]. In this work, we estimate that between 3.5 and 4.9% of the zinc used in the molten zinc bath of the HDG process is lost through the SPAs. Therefore, SPAs valorization could be an additional source of secondary zinc with potential economic benefits [27]. Zinc recovery from SPAs makes sense, since the production of special high-grade (SHG) zinc is a very energy-intensive process with a primary energy demand of 37,500 MJ and a climate change impact of 2600 kg CO_2−_ eq. per ton of primary zinc produced [28]. In addition, the zinc price was positioned in November 2020 at around 2200€ per ton, ranging during the last three years between 1650 and 2950€ per ton, thus hightlingitng the industrial interest on its recovery [29].

Technologies that enable acid recovery are spray roasting, evaporation, diffusion dialysis (DD), membrane distillation (MD), electrodialysis (ED), and membrane electrolysis (ME) [30]. Methods enabling both metal and acid recovery are ion exchange (IE)/retardation, crystallization and solvent extraction (SX) [6]. DD, ED, evaporation, precipitation, and spray roasting have been industrially implemented. Moreover, IE has been applied to recover HCl by the Metsep process. Quimigal in Portugal employed SX for the regeneration of SPAs based on the Modified Zincex Process (MZP) that allows the production of SHG zinc. SHG production from secondary sources of zinc, such as Waelz oxides, has been developed using the Zincex Process and MZP by Técnicas Reunidas since 1976, and MZP is still carried out today [31].

Table 2 summarizes previous works focused on the zinc recovery from SPAs at laboratory scale using SX and membrane-based solvent extraction (MBSX), including the innovative NDSX and emulsion pertraction (EPT) configurations, which are performed in hollow fiber membrane contactors (HFMC) [32].

HFMCs permit the non-dispersive extraction of metals by using the porous membrane to stabilize the aqueous–organic interface [44,45,46,47]. EPT and NDSX differ in the way of contacting the fluid phases and the number of contactors [48]. In NDSX, the aqueous phase circulates through the inner side of the fibers, and the organic phase circulates through the shell side [10]. On the contrary, in EPT, the organic extractant and the aqueous backextraction agent form a pseudo-emulsion that flows through the shell side, while the aqueous feed phase circulates through the membrane bore side. EPT uses a lower membrane area and achieves a higher interfacial mass transfer area for the back-extraction, although the separation of phases is needed after backextraction in order to reuse the organic phase. Therefore, in the present study, we selected the NDSX configuration to avoid any phase mixing and facilitate the scale-up to pilot plant scale.

This work is focused on the validation of the NDSX technology at a demonstrative scale in real conditions, for the selective separation and recovery of zinc from SPAs generated in the HDG process. In this context, pilot-scale experiments have been performed in an NDSX plant equipped with four hollow fiber modules and a total membrane area of 80 m^2^. Experiments have been performed with real SPAs that were supplied by an HDG manufacturer. The aims of this work are (i) the evaluation of the effect of the process operation variables to promote the selective zinc separation over iron, (ii) to obtain a stripping phase with enough quality for zinc electrowinning, and (iii) to assess the scale-up of the NDSX technology and define procedures for the industrial scale design. 

## 2. Materials and Methods 

### 2.1. Materials

Samples of SPAs were provided by GALESA, which is a hot-dip galvanizer located in Spain. Table 3 shows the chemical characterization of the SPA batch used in the pilot plant tests.

Zinc and iron concentration in SPA samples and stripping solution were measured by Microwave Plasma-Atomic Emission Spectrometry (MP-AES, Agilent, Spain). Nearly all (98.2%) of the total iron content in the SPA batch was as Fe (II), as determined by UV/Vis spectrometry (Method 1.00796.0001, Merck, Spain). Other metals that were present in low concentrations were analyzed by Inductively Coupled Plasma/Mass Spectrometry (ICP-MS, Agilent). Several studies have already determined the presence of minor metals in SPAs by atomic absorption spectroscopy [10,11]. Ion Chromatography (IC) was used to analyze chloride anions. TOC was determined in a TOC-V analyzer (Shimadzu, Spain) with external calibration. Ammonium was analyzed by alkaline distillation and acid–base titration after iron precipitation, and acidity was measured by tritation with sodium carbonate.

The composition of the organic and stripping phases were selected based on the previous expertise of the research group [25] and according to the literature review (Table 2). The extractant phase was a 50%/50% (*v*/*v*) solution of tributylphosphate (TBP) and ShellsolD70, which is an aliphatic solvent that is used to dilute TBP with the aim of reducing the viscosity of the organic stream. Tap water was used as the stripping agent.

### 2.2. Recovery of Zinc by Membrane-Based Solvent Extraction at Pilot Plant Scale

**Figure 1:**
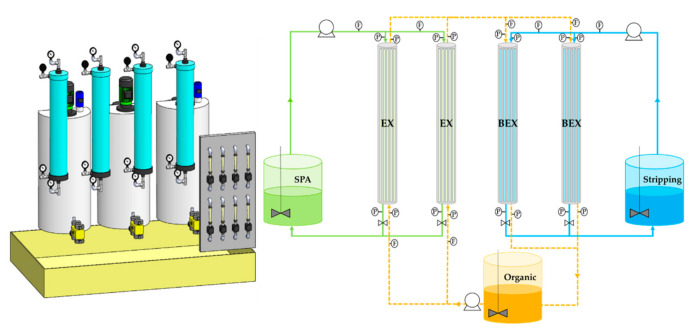
Non-dispersive solvent extraction (NDSX) pilot plant.

Figure 1 presents the scheme of the pilot plant unit used in the demonstration tests. The pilot plant integrates 4 microporous polypropylene hollow-fiber membrane contactors (3M™ Liqui-Cel ™ EXF-4 × 28 Series) whose main characteristics are summarized in Table 4. Technical information for the HFMC can be found in the 3M™ website [49].

The SPA feed phase, the organic extractant phase, and the aqueous stripping phase were allocated in three tanks. Two HF modules were used for the extraction (EX) step, in which zinc was transferred from the SPA feed phase that flowed through the inner side of the fibers to the organic extractant phase that flowed through the shell side of the module. In the two back-extraction (BEX) HF modules, zinc was backextracted by the stripping water flowing through the inner side of the membranes, while at the same time, the organic extractant was regenerated and recycled to the extraction modules again. The system is equipped with filters to prevent the entry of solids in the membrane contactors and a stage of oils and fats removal. To initiate the operation, the pneumatic pumps flowed the aqueous phases (feed and stripping) through the HF modules; next, the organic phase flow started. The hydrodynamic pressure of all streams was adjusted with the back-pressure valves located at the exit of the HF modules to achieve a minimum 0.15 bar overpressure of every aqueous stream over the organic phase flowing through the same HF module at both inlet and outlet positions of each membrane contactor. This mode of operation prevents the penetration of the organic phase into the aqueous phases and maintains the aqueous–organic interphase at the porous wall of the hydrophobic polypropylene membranes. Experiments were performed at room temperature. Two flowmeters were installed at the inlet of each HFMC for measuring the flowrate of the two inlet aqueous and organic streams. Optimal volumes of each phase and operating pressures were fixed based on preliminary experiments developed by the research group at the laboratory scale using HF contactors (Liqui-Cel Extra-Flow 2.5 in. × 8 in.) with an effective membrane area of 1.4 m^2^ [25]. Table 5 shows the experimental conditions in the pilot plant experiments conducted for the demonstration of zinc recovery by NDSX.

## 3. Results and Discussion

### 3.1. Mechanism of Zinc Extraction

The mechanism of zinc and iron extraction by TBP in chloride media has been described by several authors, as summarized by Lum et al. [38]. All authors agree on the relevance of metal chlorocomplexes, their speciation being influenced by the concentration of zinc and iron, chloride, and pH of the SPA. The software Medusa can be used to estimate the chlorocomplexes distribution in the system [50]. In the present study, the species present in the feed solution, as predicted by Medusa, are Cl^−^, ZnCl_4_^2−^, ZnCl^3−^, ZnCl_2_, ZnCl^+^, Zn^2+^, Fe^2+^ and FeCl^+^, of which ZnCl_4_^2−^ and Fe^2+^ are the predominant ones. This confirms that the high ionic strength of the SPA leads to the formation of chlorocomplexes with a high stoichiometric coefficient of chlorine [51]. The speciation of zinc as negative chlorocomplexes and iron as positive species opens the door to the selective separation of zinc, as TBP extracts negative species preferentially. Therefore, the reaction equilibria for the extraction of zinc is described by reaction (1),

(1) $$Zn^{2+}+4Cl^{-}+2H^{+}+4\bar{TBP}⇌\;\bar{H_{2}ZnCl_{4}\;4TBP}$$

where zinc mass transfer is accompanied by chloride and protons. When the acidity of the SPAs decreases, reactions (2) and (3) may become relevant,

(2) $$Zn^{2+}+3Cl^{-}+H^{+}+3\bar{TBP}⇌\;\bar{HZnCl_{3}\;3TBP}$$

(3) $$Zn^{2+}+2Cl^{-}+2\bar{TBP}⇌\;\bar{ZnCl_{2}\;2TBP}$$

### 3.2. Selective Zinc Extraction at Pilot Plant Scale

#### 3.2.1. Selective Zinc Separation in Pilot Plant Experiments

**Figure 2:**
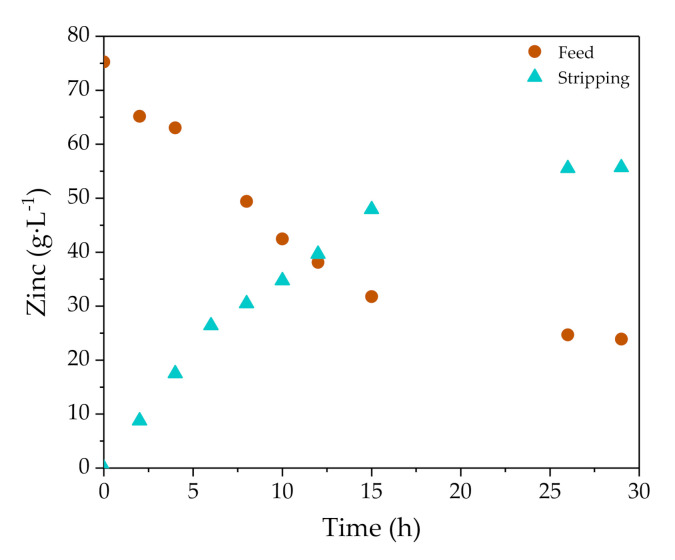
Zinc concentration evolution with time in the feed and stripping phases using NDSX technology in a representative pilot scale test (EXP III).

**Figure 3:**
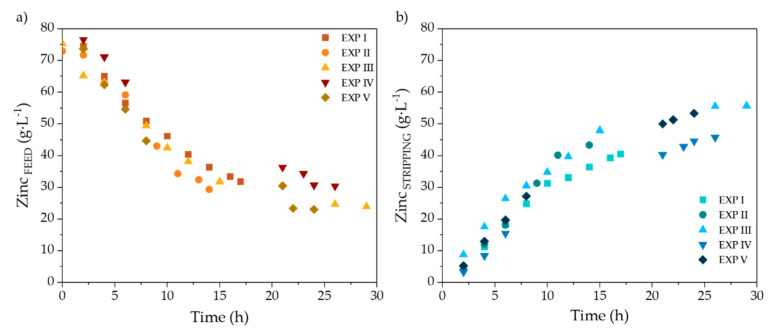
Zinc concentration in the feed phase (**a**) and in the stripping phase (**b**) with experimental time for EXP I–V.

Figure 2 shows the evolution of zinc concentration in the feed and stripping phases during EXP III that was performed in conditions as defined in Table 5. Zinc concentration in the feed phase decreased from 75.2 to 23.9 g·L^−1^, while the stripping phase achieved 55.7 g·L^−1^ of zinc. The process was carried out for 29 h, although zinc mass transfer was very slow in the final period, as chemical equilibrium conditions were being approached. Figure 3 shows the evolution of zinc concentration in the feed and stripping phases in the whole set of five experiments (EXP I-V). Experimental points are reasonably overlapped, showing that the performance of the organic phase for EX/BEX was maintained along the five consecutive cycles. The experimental conditions shown in Table 5 determine the differences in reproducibility of results in Figure 3.

**Figure 4:**
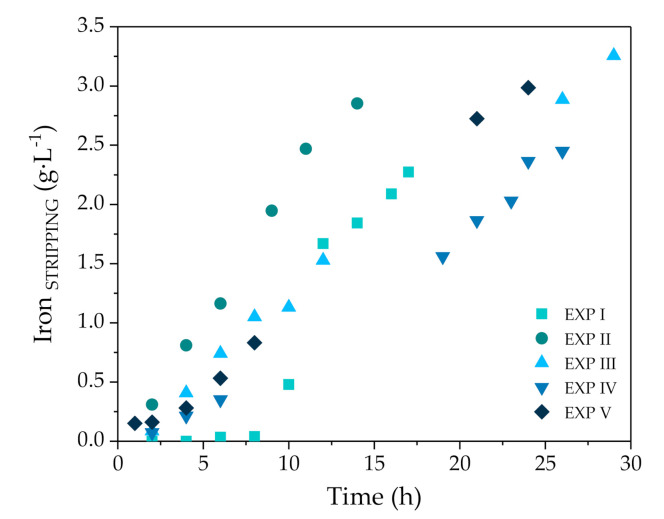
Iron concentration with experimental time in the stripping (S) phase using NDSX technology for EXP I–V.

Most of the iron of the stripping phase is present as Fe^3+^, which is in accordance with previous literature reporting the low selectivity of zinc solvation extractants over Fe^3+^ and the much higher selectivity of reagents over Fe^2+^ [35]. The iron concentration in the stripping phase at the end of EXP I-V was between 2.2 and 3.3 g·L^−1^ (Figure 4). This performance is translated into a high selectivity of the Zn/Fe separation. For the representative EXP III, the selectivity of zinc over iron in the stripping phase can be calculated as the relation of the empirical initial fluxes: J_Zn_/J_Fe_ = 1.09 × 10^−1^ mol Zn·m^−2^·h^−1^/4.68 × 10^−3^ mol Fe·m^−2^·h^−1^ = 23.2.

**Figure 5:**
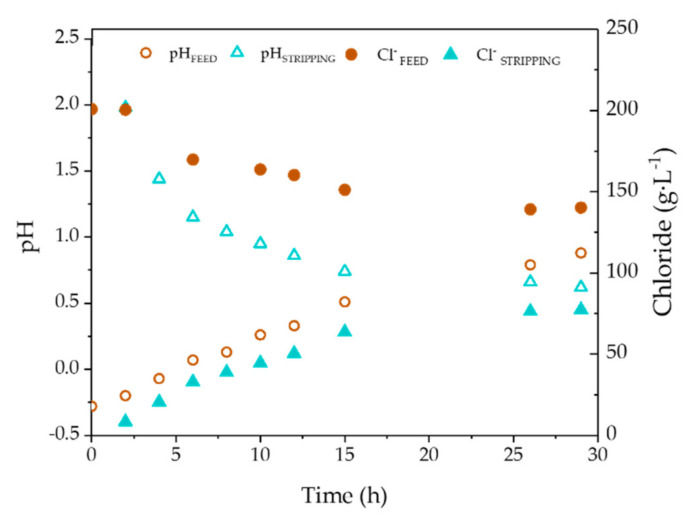
Evolution of pH and chloride concentration with experimental time in the feed and stripping phases using NDSX technology in a representative experiment (EXP III).

Figure 5 presents the performance of chloride and pH in the feed and stripping phases in EXP III. Zinc extraction/backextraction by TBP involves the participation of both species, as it is depicted in reactions (1)–(3). As a result of the chloride and protons mass transfer, the water used as the stripping phase becomes more acidic and chloride enriched. It can be seen that at the end of EXP III, the pHs of the feed and stripping phases were equilibrated, which is a factor that explains the interruption of zinc transfer observed at *t* = 26 h (Figure 3).

Table 6 presents the chemical characterization of the stripping phase at the end of each experiment. The analytical signal of other minor metals was below the quantification limit of the analytical method, proving the high selectivity of the separation process.

Considering the overall objective of the zinc separation process, the stripping phase should increase its zinc concentration as much as possible, while its iron concentration should be minimized. Since the mass transfer of zinc is limited by the equilibrium, and the iron mass transfer shows a linear increase with time, the separation batch should be stopped once the zinc concentration in the stripping phase achieves its maximum value in order to avoid further iron transfer. The small variability in the values of zinc and iron concentration at the end of the tests (Table 6) can be assigned to the differences in the duration and the volumes of feed and stripping phase used in each experimental run (Table 5). Even taking into consideration these variabilities, the final molar ratios of zinc over iron in the stripping phase is within a narrow range of 13 to 15.9 mol Zn/mol Fe in the whole set of five experiments EXP I-V.

#### 3.2.2. Analysis of Zinc Separation Mechanism

**Figure 6:**
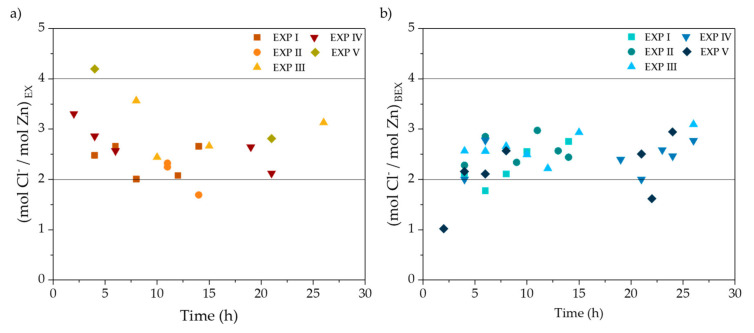
Molar ratio of chloride/zinc during the extraction (**a**) and backextraction (**b**) for EXP I-V.

As mentioned in Section 3.1, zinc extraction by TBP is accompanied by chloride and protons, as Figure 2 and Figure 5 show. Next, Figure 6 presents the chloride to zinc molar ratio in the feed and stripping phases along the duration of EXP I-V. Most values of the Cl/Zn ratio in the SPA feed phase are comprised between 4 and 2, being closer to 4 in the first part of the extraction run. Those values are in good agreement with reactions (1) to (3) and Medusa calculations, which predicted that ZnCl_4_^2−^ was the predominant species in the feed SPA. Therefore, during the first part of the extraction cycle, the chloride to zinc ratio should be close to 4, according to reaction (1). Nevertheless, ZnCl_3_^−^ and ZnCl_2_ species that are also present in the feed SPAs are extracted according to reactions (2) and (3), which is a factor that reduces the Zn/Cl ratio of extraction. In the stripping phase, the initial chloride and proton concentration are much lower than in the feed, and the speciation of zinc chlorocomplexes differs from the feed phase. However, in the conditions of the stripping phase at the end of the extraction/backextraction cycle, ZnCl_4_^2−^ is also the major zinc chlorocomplex.

#### 3.2.3. Scale-Up from Laboratory to Pilot Scale

We have selected the mass flux of zinc as a scale-up parameter for the design of the NDSX process for zinc selective separation from SPAs generated in the HDG process. Therefore, the zinc mass flux (*J*) has been calculated from extraction and backextraction data, using Equations (4) and (5),

(4) $$J_{EX}=\frac{\left({\left[Zn\right]}_{t-1}-{\left[Zn\right]}_{t}\right)\times V_{F}}{A_{EX}\times \Delta t}$$

(5) $$J_{BEX}=\frac{\left({\left[Zn\right]}_{t}-{\left[Zn\right]}_{t-1}\right)\times V_{S}}{A_{BEX}\times \Delta t}$$

**Figure 7:**
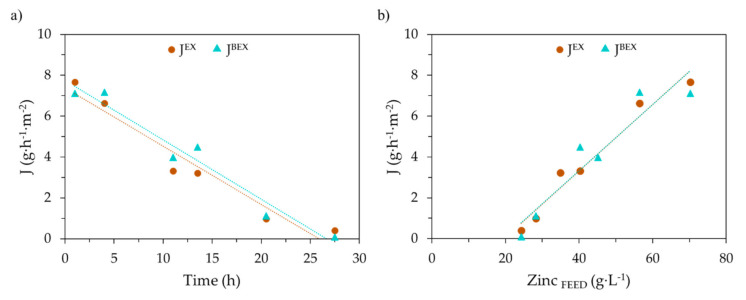
Zn flux, calculated from extraction (*J_EX_*) and backextraction (*J_BEX_*) data of EXP III. (**a**) *J_EX_, J_BEX_* evolution with time; (**b**) *J_EX_, J_BEX_* as a function of zinc concentration in the feed phase.

Figure 7a presents the evolution with time of *J_EX_* and *J_BEX_*. Similarly, Figure 7b shows the relation between the mass flux of Zn with the zinc concentration in the feed phase.

Zinc flux values in Figure 7a calculated form EX and BEX data show a linear evolution with time. The dependence of zinc EX and BEX fluxes with zinc concentration in the feed phase also fits a linear function, with higher fluxes at increasing zinc concentration. *J_EX_* and *J_BEX_* values are very similar; therefore, zinc accumulation in the organic phase would be negligible. This behavior was expected, as the driving force for zinc transfer depends on the gradient of zinc concentration between the feed and the stripping phases. The phenomenological process modeling of the system under study is hindered by the complexity of the solvation mechanism involved in the zinc EX/BEX reactions and the evolution in the predominant zinc chlorocomplexes in the feed phase due to its changing chemical composition.

**Figure 8:**
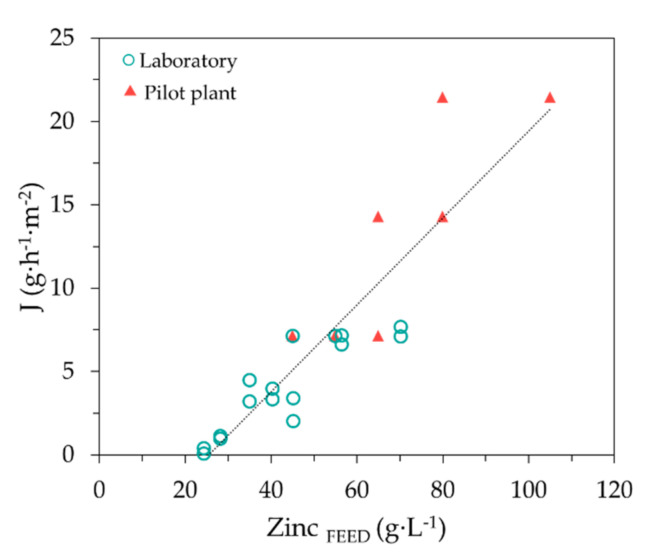
Zinc flux *vs*. zinc concentration in the feed phase. *J_EX_* and *J_BEX_* calculated from data of EXP III in this study (▲), and form bench scale data (○) reported by Laso et al. [25].

Figure 8 plots together zinc *J_EX_* and *J_BEX_* fluxes obtained in the present pilot plant work, with data previously reported by our group working in a bench-scale experimental system provided with 2 HFMCs, each one with 1.4 m^2^ of membrane area [25]. In that previous study, the SPA batch was more acidic (40.11 ± 1.46 g HCl·L^−1^) and with higher zinc concentration (122 ± 3 g Zn^2+^·L^−1^). All together, bench scale and pilot scale data can be fitted to the same linear equation, with a reasonably good regression parameter. The linear fitting depicted in Figure 8 allows obtaining the following zinc mass transfer flux equation (Equation (6)),

(6) $$J\;\left(g\;h^{-1}\;m^{-2}\right)=0.26\;\left(L\;h^{-1}\;m^{-2}\right)\times C\;\left(g\;L^{-1}\right)-6.62\;\left(g\;h^{-1}\;m^{-2}\right).$$

The flux data shown in Figure 8 and Equation (6) confirm that the modularity of membrane systems enhances the easy scale-up of membrane-based separation systems. Finally, Equation (6) is proposed to define the mass transfer of zinc as a function of zinc concentration that is needed for dimensioning the NDSX system for the selective separation of zinc from spent pickling acids generated in hot-dip galvanizing facilities.

## 4. Conclusions

This work is aimed at developing a key enabling technology to move the galvanizing sector toward a sustainable use of metallic resources. In the frame of the LIFE2ACID project, we propose the selective recovery of zinc from SPAs generated in HDG facilities by the integration of membrane based non-dispersive solvent extraction (NDSX) and electrowinning (EW). The present study deals with the demonstration of the NDSX at the pilot scale, using a membrane-based solvent extraction plant with 80 m^2^ of total membrane area and TBP as a selective extractant to treat industrial SPAs with relevant zinc and iron content (71.7 ± 4.3 g Zn·L^−1^ and 82.9 ± 5.0 g Fe·L^−1^). Zinc was recovered as metal dissolved in aqueous solution with a concentration of 55.7 g Zn·L^−1^ and reduced iron content of 3.2 g Fe·L^−1^. Favorably, other minor metals that are present in the SPAs were not transferred to the zinc-enriched stripping solution. The stable performance of the pilot plant in five consecutive batch extraction/backextraction cycles showed that the operation of the NDSX systems achieves the adequate regeneration and chemical stability of the organic extractant. Zinc extraction mechanism and mass transfer fluxes were satisfactorily correlated with previous laboratory-scale literature data. Therefore, this study defines the function that relates zinc mass transfer flux with the zinc concentration in the SPAs that is needed for design and scale-up purposes of the technology toward its prototype on-site demonstration in real galvanizing facilities. The presence of small iron concentration in the purified zinc liquor is not expected to prevent the EW recovery of secondary zinc with purity >99.5%. However, iron transfer should be minimized for zinc purities >99.9%. Future research will be focused on defining the operation conditions needed to avoid iron (II) oxidation, in order to prevent the iron (III) transfer that was observed in the present pilot plant demonstration study. Our study demonstrates the potentiality of NDSX technology for recovering zinc from residual SPAs generated in HDG, at a pilot scale never tested before, which could be reused either by galvanizers or as supply for other secondary zinc markets.

## Figures and Tables

**Table 1:** Composition of real spent pickling acids based in HCl.

Zn (g·L^−1^)	Fe^TOTAL^ (g·L^−1^)	Fe^2+^ (g·L^−1^)	Fe^3+^ (g·L^−1^)	HCl (g·L^−1^)	H^+^ (mol·L^−1^)	Cl^−^ (g·L^−1^)	pH	Ref.
26.1	137.5	-	-	17.9	-	-	-	[11]
82	-	96	-	-	-	227	≈0	[10,12,13,14]
<130	<100	-	-	10%	-	-	-	[15]
20–120	100–130	-	-	36.5–219	-	-	-	[2]
70.2	92.2	-	-	9.1	-	-	-	[16]
5–150	8–150	-	-	10–80	-	-	-	[1,9]
<150	-	-	-	3.6%	-	-	-	[17]
<120	<204	-	-	-	-	-	-	[18,19]
78.5	89.4	-	-	237	-	-	-	[20]
-	-	35–45	-	-	-	-	-	[7]
100–120	-	30–32	1–2	90–100	-	230–250	-	[21]
129.3	58.8	-	-	78	-	-	-	[22,23]
134	-	74	<1	36	-	300	-	[24]
122 ± 3	95.6 ± 3	92.6 ± 2	3 ± 2	-	1.1 ± 0.04	301 ± 3	-	[25]

**Table 2:** Literature review of zinc recovery from spent pickling acids (SPAs) by solvent extraction (SX) and membrane-based solvent extraction (MBSX).

Method/Configuration	Extractant	Diluent	Stripping Agent	Ref.
SX	Cyanex 921/Cyanex 923/Cyanex 302/TBP/ALAMINE336	Kerosene	Water	[33]
	TBP/ DEHPA/HOE F 2562/ ALIQUAT 336/ALAMINE 304-308-310-336/CYANEX 301	Kerosene Exxsol D220/230	Water/HCl	[34]
	TBP	Exxsol D 220/230	Water	[35]
	Alamine 336	m-Xylene	Na_2_CO_3_ (aq.)	[36]
	TBP/Cyanex 272/Cyanex 301/Cyanex 302	Kerosene	Water/HCl	[37]
	TBP/Hostarex A226, A324, and A327	Kerosene	Water	[17]
	TBP/Cyanex 301/Cyanex 272	Exxsol D-80	H_2_SO_4_	[16]
	Cyphos IL 101	Toluene	H_2_SO_4_	[18]
	1-(3-pyridyl)undecan-1-one	Decan-1-ol	Na_2_SO_4_	[19]
	TBP	ShellSol 2046	Water	[38]
	Tri-iso-octyl amine (TiOA)/tris(2-ethylhexyl) amine (TEHA)	Di-(2-ethylhexyl) phosphoric acid (DEHPA)	Water/H_2_SO_4_	[21]
	TBP, D2EHPA	Shellsol 2046	Water	[39]
	TBP	-	Oxalic acid	[40]
	TBP/D2EHPA	Kerosene	Water/HCl	[3]
	Cyanex 923/TEHA	Kerosene	Diluted ammonia	[41]
	Aliquat 336	Sulfonated kerosene	Water/H_2_SO_4_/NaOH/NH_3_·H_2_O	[42]
NDSX	TBP	-	Water	[13]
	TBP/Cyanex 272/DEHPA	Kerosene	Water/H_2_SO_4_	[2]
	TBP	Shellsol 2046	Water	[24]
	TBP	-	Water	[12]
EPT	TBP	-	Water	[14]
	1-(3-pyridyl)undecan-1-one oxime/TBP	Toluene, ShellSol D70 and decan-1-ol	Na_2_SO_4_/Water	[27]
NDSX/EPT	TBP	Shellsol D70	Water	[25]
SX and Polymer inclusion membranes (PIM)	Cyphos IL 101/ Cyphos IL 104	Toluene	H_2_SO_4_	[43]
EPT and DD	TBP	-	Water/NaOH	[20]
NDSX and ED	TBP	-	Water	[44]

**Table 3:** Composition of spent pickling acids.

pH	-	≈0
Free acidity (HCl)	g·L^−1^	13.4 ± 3.4
Chloride		212.6 ± 41.6
Zinc		71.7 ± 4.3
Iron		82.9 ± 5.0
Manganese		0.91 ± 0.01
Chromium		0.07 ± 0.01
Aluminium		0.04 ± 0.01
Molybdenum		0.05 ± 0.01
Nickel		<0.0025
Tin		
Bismuth		
Lead		
Wolfram		
NH_4_^+^		0.050
Total organic carbon (TOC)		2.93

**Table 4:** Characteristics of the hollow fiber membrane contactors.

Characteristic	Unit	Pilot Plant (Liqui-Cel Extra-Flow 4 in. × 28 in.)
Cartridge dimensions (D × L)	cm	11.6 × 88.9
Num. fibers	-	36,675
Effective membrane surface area	m^2^	20
Effective length	m	0.789
Fiber type	-	×50
Fiber material	-	Polypropylene
Inner diameter (fibers)	µm	220
Wall thickness (fibers)	µm	80
Porosity	%	40

**Table 5:** Experimental conditions used in the non-dispersive solvent extraction (NDSX) pilot plant tests.

		EXP.				
Exp. Conditions	Units	I	II	III	IV	V
Feed volume	L	69	57	61	91	71
Organic volume		83	86	76	76	75
Stripping volume		97	59	65	125	94
Feed phase (SPA) flowrate	L·h^−1^	200	50	50	50	100
Organic extractant phase flowrate		200	150	150	150	200
Stripping phase (water) flowrate		200	50	100	100	150
Hollow fiber membrane contactors	-	4				
Extraction membrane area	m^2^	40				
Backextraction membrane area		40				

**Table 6:** Chemical characterization of the final stripping phase in EXP I-V.

		EXP.				
	Units	I	II	III	IV	V
Zinc^TOTAL^	g·L^−1^	40.5	43.3	55.7	45.7	53.3
Iron^TOTAL^		2.3	2.9	3.3	2.4	3.0
Chloride (Cl^−^)		55.9	58.6	77.4	59.3	67.4
Free acidity (H^+^)		0.173	0.223	0.272	0.416	0.128
Manganese		0.026	0.010	0.047	0.040	0.018
Molybdenum		0.003	0.005	0.008	0.006	0.001
Tin		0.001	0.002	0.002	0.002	0.001
pH		0.80	0.69	0.62	0.73	0.72
